# (4*E*)-*N*-[(2-Bromo­phen­yl)meth­oxy]-1,3-dimethyl-2,6-diphenyl­piperidin-4-imine

**DOI:** 10.1107/S1600536812028887

**Published:** 2012-06-30

**Authors:** Chennan Ramalingan, Seik Weng Ng, Edward R. T. Tiekink

**Affiliations:** a Centre for Nanotechnology, Department of Chemistry, Kalasalingam University, Krishnankoil 626 126, Tamilnadu, India; b Department of Chemistry, University of Malaya, 50603 Kuala Lumpur, Malaysia; c Chemistry Department and Faculty of Science, King Abdulaziz University, PO Box 80203 Jeddah, Saudi Arabia

## Abstract

In the title compound, C_26_H_27_BrN_2_O, the piperidine ring has a chair conformation and all ring substituents occupy equatorial positions, apart from the double-bonded N atom, which occupies a bis­ectional position. The dihedral angle formed between the phenyl rings is 61.18 (19)°, and the phenyl rings form dihedral angles of 49.78 (19) and 69.2 (18)° with the bromo­benzene ring. The latter is coplanar with the meth­oxy(methyl­idene)amine fragment [N—O—C—C torsion angle = −171.7 (2)°]. Linear supra­molecular chains, approximately along [112], sustained by C—H⋯π inter­actions, feature in the crystal packing.

## Related literature

For the biological activity of mol­ecules having a 2,6-diaryl­piperidine core, see: Ramachandran *et al.* (2011 ▶); Ramalingan *et al.* (2004 ▶). For the structure of the chloro derivative, see: Ramalingan *et al.* (2012 ▶). For the synthesis, see: Ramalingan *et al.* (2006 ▶).
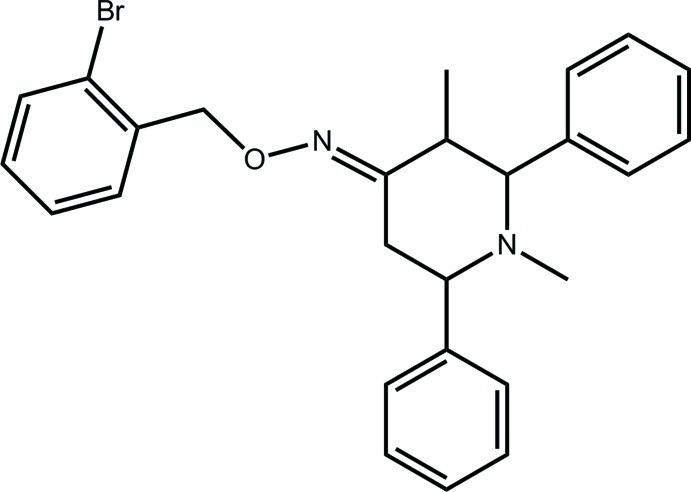


## Experimental

### 

#### Crystal data

C_26_H_27_BrN_2_O
*M* *_r_* = 463.41
Triclinic, 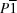

*a* = 10.4425 (6) Å
*b* = 11.2544 (6) Å
*c* = 11.7035 (6) Å
α = 106.635 (5)°
β = 104.289 (5)°
γ = 113.558 (5)°
*V* = 1101.14 (14) Å^3^
*Z* = 2
Mo *K*α radiation
μ = 1.89 mm^−1^
*T* = 100 K
0.30 × 0.25 × 0.20 mm

#### Data collection

Agilent SuperNova Dual diffractometer with an Atlas detector
Absorption correction: multi-scan (*CrysAlis PRO*; Agilent, 2012 ▶) *T*_min_ = 0.705, *T*_max_ = 1.000
16609 measured reflections
5097 independent reflections
4176 reflections with *I* > 2σ(*I*)
*R*_int_ = 0.060

#### Refinement

*R*[*F* ^2^ > 2σ(*F* ^2^)] = 0.047
*wR*(*F* ^2^) = 0.109
*S* = 1.08
5097 reflections
271 parameters
H-atom parameters constrained
Δρ_max_ = 0.80 e Å^−3^
Δρ_min_ = −0.47 e Å^−3^

### 

Data collection: *CrysAlis PRO* (Agilent, 2012 ▶); cell refinement: *CrysAlis PRO*; data reduction: *CrysAlis PRO*; program(s) used to solve structure: *SHELXS97* (Sheldrick, 2008 ▶); program(s) used to refine structure: *SHELXL97* (Sheldrick, 2008 ▶); molecular graphics: *ORTEP-3 for Windows* (Farrugia, 1997 ▶) and *DIAMOND* (Brandenburg, 2006 ▶); software used to prepare material for publication: *publCIF* (Westrip, 2010 ▶).

## Supplementary Material

Crystal structure: contains datablock(s) global, I. DOI: 10.1107/S1600536812028887/bt5957sup1.cif

Structure factors: contains datablock(s) I. DOI: 10.1107/S1600536812028887/bt5957Isup2.hkl

Supplementary material file. DOI: 10.1107/S1600536812028887/bt5957Isup3.cml

Additional supplementary materials:  crystallographic information; 3D view; checkCIF report

## Figures and Tables

**Table 1:** Hydrogen-bond geometry (Å, °) *Cg*1 is the centroid of the C21–C26 benzene ring.

*D*—H⋯*A*	*D*—H	H⋯*A*	*D*⋯*A*	*D*—H⋯*A*
C4—H4⋯*Cg*1^i^	0.95	2.77	3.626 (4)	150

Symmetry code: (i) 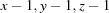.

**Fig. 1.**
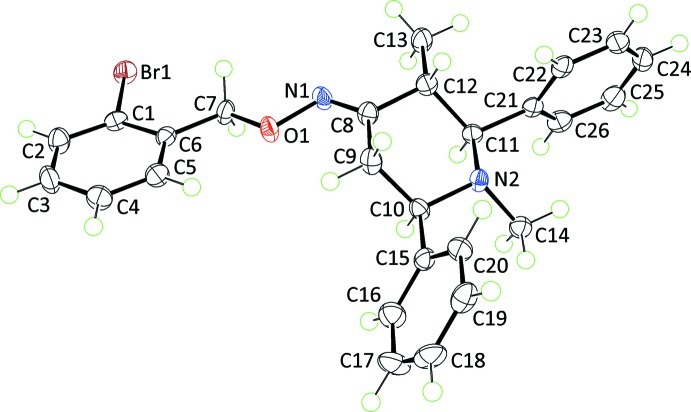
The molecular structure of (I) showing the atom-labelling scheme and displacement ellipsoids at the 70% probability level.

**Fig. 2.**
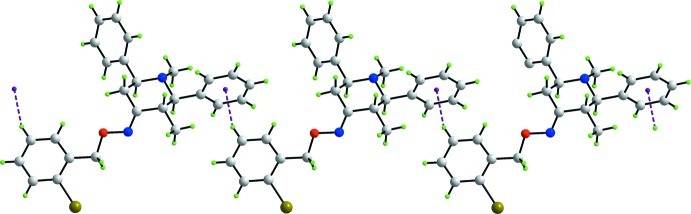
A view of the supramolecular chain in (I) sustained by C—H···π interactions, shown as purple dashed lines.

**Fig. 3.**
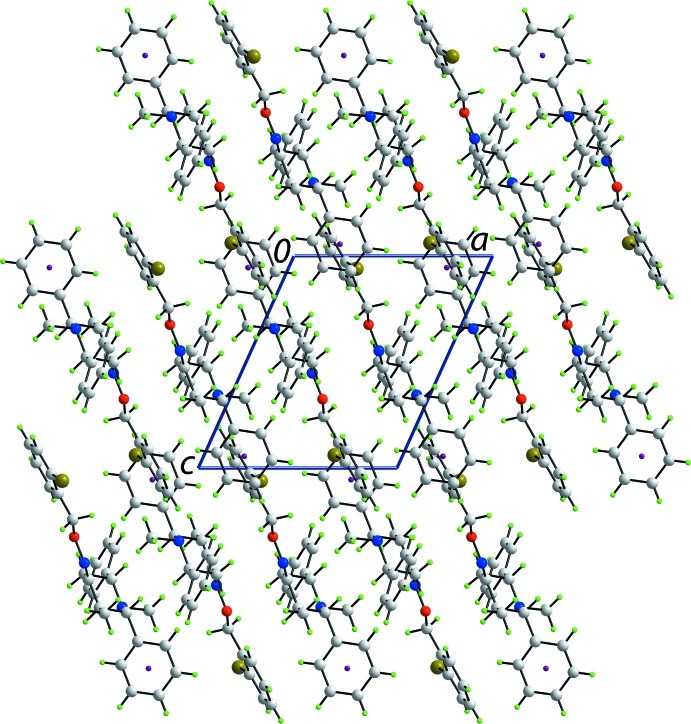
A view in projection down the b axis of the unit-cell contents for (I), showing the stacking of layers. The C—H···π interactions are shown as purple dashed lines.

## supplementary crystallographic information

### Comment

The original synthesis (Ramalingan *et al.*, 2006) of the title compound, (I), was motivated by the diverse range of molecules possessing a 2,6-diarylpiperidine core that exhibit potent biological activities (Ramachandran *et al.*, 2011; Ramalingan *et al.*, 2004). Herein, the crystal and molecular structure of (I) is described.

In (I), Fig. 1, the piperidine ring has a chair conformation and all ring-substituents bound to C occupy equatorial positions, as found for the chloro derivative (Ramalingan *et al.*, 2012), but the the double bonded N atom occupies a bisectional position. The dihedral angle formed between the C15–C20 and C21–C26 phenyl rings is 61.18 (19)°, and each forms a dihedral angle of 49.78 (19) and 69.2 (18)°, respectively, with the bromobenzene ring, which occupies a position co-planar to the methoxy(methylidene)amine residue as seen in the N1—O1—C7—C6 torsion angle of -171.7 (2)°. This is in contrast to the orthogonal disposition in the chloro derivative (Ramalingan *et al.*, 2012). The conformation about the imine C8═N1 bond [1.281 (4) Å] is *E*.

In the crystal packing, linear supramolecular chains are formed *via* C—H···π interactions, Fig. 2 and Table 1. These assemble into layers parallel to (1 0 1) and stack without specific intermolecular interactions between the chains, Fig. 3.

### Experimental

For full details of the synthesis, refer to Ramalingan *et al.* (2006). Re-crystallization was performed by slow evaporation of an ethanolic solution of (I) which afforded colourless crystals. *M*.pt: 378–378 K.

### Refinement

Carbon-bound H-atoms were placed in calculated positions [C—H = 0.95–0.99 Å, *U*_iso_(H) = 1.2–1.5*U*_eq_(C)] and were included in the refinement in the riding model approximation. Owing to poor agreement, a reflection, *i.e*. (-6 4 9), was omitted from the final refinement.

### Crystal data

**Table:**

C_26_H_27_BrN_2_O	*Z* = 2
*M**_r_* = 463.41	*F*(000) = 480
Triclinic, *P*1	*D*_x_ = 1.398 Mg m^−^^3^
Hall symbol: -P 1	Mo *K*α radiation, λ = 0.71073 Å
*a* = 10.4425 (6) Å	Cell parameters from 3935 reflections
*b* = 11.2544 (6) Å	θ = 2.2–27.5°
*c* = 11.7035 (6) Å	µ = 1.89 mm^−^^1^
α = 106.635 (5)°	*T* = 100 K
β = 104.289 (5)°	Prism, colourless
γ = 113.558 (5)°	0.30 × 0.25 × 0.20 mm
*V* = 1101.14 (14) Å^3^	

### Data collection

**Table:**

Agilent SuperNova Dual diffractometer with an Atlas detector	5097 independent reflections
Radiation source: SuperNova (Mo) X-ray Source	4176 reflections with *I* > 2σ(*I*)
Mirror monochromator	*R*_int_ = 0.060
Detector resolution: 10.4041 pixels mm^-1^	θ_max_ = 27.6°, θ_min_ = 2.2°
ω scan	*h* = −13→13
Absorption correction: multi-scan (*CrysAlis PRO*; Agilent, 2012)	*k* = −14→14
*T*_min_ = 0.705, *T*_max_ = 1.000	*l* = −15→15
16609 measured reflections	

### Refinement

**Table:**

Refinement on *F*^2^	Primary atom site location: structure-invariant direct methods
Least-squares matrix: full	Secondary atom site location: difference Fourier map
*R*[*F*^2^ > 2σ(*F*^2^)] = 0.047	Hydrogen site location: inferred from neighbouring sites
*wR*(*F*^2^) = 0.109	H-atom parameters constrained
*S* = 1.08	*w* = 1/[σ^2^(*F*_o_^2^) + (0.0415*P*)^2^ + 0.7452*P*] where *P* = (*F*_o_^2^ + 2*F*_c_^2^)/3
5097 reflections	(Δ/σ)_max_ = 0.001
271 parameters	Δρ_max_ = 0.80 e Å^−^^3^
0 restraints	Δρ_min_ = −0.47 e Å^−^^3^

### Special details

**Table:**

Geometry. All e.s.d.'s (except the e.s.d. in the dihedral angle between two l.s. planes) are estimated using the full covariance matrix. The cell e.s.d.'s are taken into account individually in the estimation of e.s.d.'s in distances, angles and torsion angles; correlations between e.s.d.'s in cell parameters are only used when they are defined by crystal symmetry. An approximate (isotropic) treatment of cell e.s.d.'s is used for estimating e.s.d.'s involving l.s. planes.
Refinement. Refinement of *F*^2^ against ALL reflections. The weighted *R*-factor *wR* and goodness of fit *S* are based on *F*^2^, conventional *R*-factors *R* are based on *F*, with *F* set to zero for negative *F*^2^. The threshold expression of *F*^2^ > σ(*F*^2^) is used only for calculating *R*-factors(gt) *etc*. and is not relevant to the choice of reflections for refinement. *R*-factors based on *F*^2^ are statistically about twice as large as those based on *F*, and *R*- factors based on ALL data will be even larger.

### Fractional atomic coordinates and isotropic or equivalent isotropic displacement parameters (Å^2^)

**Table:**

	*x*	*y*	*z*	*U*_iso_*/*U*_eq_	
Br1	0.34353 (4)	0.50273 (3)	0.06043 (3)	0.01907 (11)	
O1	0.5312 (2)	0.3306 (2)	0.32446 (19)	0.0175 (5)	
N1	0.6428 (3)	0.4466 (3)	0.4478 (2)	0.0167 (5)	
N2	0.9330 (3)	0.3545 (2)	0.6563 (2)	0.0136 (5)	
C1	0.2741 (3)	0.3069 (3)	0.0280 (3)	0.0144 (6)	
C2	0.1471 (4)	0.2010 (3)	−0.0865 (3)	0.0195 (7)	
H2	0.0955	0.2262	−0.1457	0.023*	
C3	0.0963 (4)	0.0579 (3)	−0.1135 (3)	0.0217 (7)	
H3	0.0098	−0.0158	−0.1919	0.026*	
C4	0.1719 (4)	0.0225 (3)	−0.0257 (3)	0.0201 (7)	
H4	0.1381	−0.0757	−0.0448	0.024*	
C5	0.2961 (4)	0.1291 (3)	0.0890 (3)	0.0186 (6)	
H5	0.3452	0.1032	0.1491	0.022*	
C6	0.3511 (3)	0.2740 (3)	0.1189 (3)	0.0140 (6)	
C7	0.4854 (3)	0.3926 (3)	0.2434 (3)	0.0169 (6)	
H7A	0.5713	0.4472	0.2236	0.020*	
H7B	0.4564	0.4597	0.2892	0.020*	
C8	0.7003 (3)	0.4035 (3)	0.5256 (3)	0.0155 (6)	
C9	0.6668 (3)	0.2537 (3)	0.4985 (3)	0.0175 (6)	
H9A	0.5930	0.1878	0.4064	0.021*	
H9B	0.6199	0.2216	0.5558	0.021*	
C10	0.8151 (3)	0.2486 (3)	0.5232 (3)	0.0143 (6)	
H10	0.8553	0.2735	0.4591	0.017*	
C11	0.9663 (3)	0.5032 (3)	0.6808 (3)	0.0134 (6)	
H11	1.0066	0.5283	0.6168	0.016*	
C12	0.8204 (3)	0.5149 (3)	0.6595 (3)	0.0156 (6)	
H12	0.7814	0.4915	0.7247	0.019*	
C13	0.8565 (4)	0.6682 (3)	0.6841 (3)	0.0202 (7)	
H13A	0.7629	0.6732	0.6726	0.030*	
H13B	0.8958	0.6943	0.6218	0.030*	
H13C	0.9338	0.7351	0.7735	0.030*	
C14	1.0753 (3)	0.3510 (3)	0.6710 (3)	0.0163 (6)	
H14A	1.0553	0.2535	0.6543	0.024*	
H14B	1.1532	0.4181	0.7602	0.024*	
H14C	1.1122	0.3791	0.6084	0.024*	
C15	0.7794 (3)	0.0967 (3)	0.4983 (3)	0.0163 (6)	
C16	0.7581 (4)	0.0031 (3)	0.3793 (3)	0.0217 (7)	
H16	0.7705	0.0358	0.3142	0.026*	
C17	0.7188 (4)	−0.1383 (3)	0.3541 (3)	0.0276 (8)	
H17	0.7037	−0.2016	0.2718	0.033*	
C18	0.7016 (4)	−0.1870 (3)	0.4482 (3)	0.0245 (7)	
H18	0.6760	−0.2831	0.4313	0.029*	
C19	0.7219 (4)	−0.0947 (3)	0.5671 (3)	0.0215 (7)	
H19	0.7103	−0.1276	0.6322	0.026*	
C20	0.7592 (3)	0.0458 (3)	0.5917 (3)	0.0183 (6)	
H20	0.7711	0.1080	0.6731	0.022*	
C21	1.0900 (3)	0.6096 (3)	0.8177 (3)	0.0139 (6)	
C22	1.0586 (4)	0.6152 (3)	0.9273 (3)	0.0157 (6)	
H22	0.9584	0.5521	0.9163	0.019*	
C23	1.1716 (4)	0.7117 (3)	1.0529 (3)	0.0202 (7)	
H23	1.1477	0.7146	1.1266	0.024*	
C24	1.3185 (4)	0.8035 (3)	1.0709 (3)	0.0220 (7)	
H24	1.3960	0.8684	1.1567	0.026*	
C25	1.3518 (4)	0.8000 (3)	0.9626 (3)	0.0226 (7)	
H25	1.4522	0.8631	0.9741	0.027*	
C26	1.2381 (4)	0.7042 (3)	0.8374 (3)	0.0186 (6)	
H26	1.2617	0.7031	0.7639	0.022*	

### Atomic displacement parameters (Å^2^)

**Table:**

	*U*^11^	*U*^22^	*U*^33^	*U*^12^	*U*^13^	*U*^23^
Br1	0.02166 (19)	0.01454 (16)	0.02196 (17)	0.01032 (14)	0.00659 (13)	0.00942 (12)
O1	0.0184 (12)	0.0116 (10)	0.0135 (10)	0.0054 (9)	−0.0008 (9)	0.0035 (8)
N1	0.0165 (14)	0.0104 (12)	0.0142 (12)	0.0046 (11)	0.0012 (11)	0.0016 (10)
N2	0.0120 (13)	0.0097 (12)	0.0143 (12)	0.0037 (10)	0.0018 (10)	0.0049 (10)
C1	0.0162 (16)	0.0123 (14)	0.0195 (15)	0.0084 (13)	0.0095 (13)	0.0094 (12)
C2	0.0195 (17)	0.0192 (16)	0.0176 (15)	0.0092 (14)	0.0044 (13)	0.0089 (13)
C3	0.0189 (17)	0.0183 (16)	0.0154 (15)	0.0061 (14)	−0.0003 (13)	0.0027 (13)
C4	0.0206 (18)	0.0136 (15)	0.0223 (16)	0.0075 (14)	0.0070 (14)	0.0064 (13)
C5	0.0219 (17)	0.0173 (16)	0.0189 (15)	0.0120 (14)	0.0068 (13)	0.0090 (13)
C6	0.0125 (15)	0.0150 (15)	0.0153 (14)	0.0076 (13)	0.0060 (12)	0.0064 (12)
C7	0.0143 (16)	0.0144 (15)	0.0180 (15)	0.0073 (13)	0.0009 (13)	0.0070 (12)
C8	0.0132 (15)	0.0135 (15)	0.0165 (15)	0.0050 (13)	0.0043 (12)	0.0065 (12)
C9	0.0165 (16)	0.0108 (14)	0.0176 (15)	0.0037 (13)	0.0020 (13)	0.0056 (12)
C10	0.0166 (16)	0.0112 (14)	0.0118 (14)	0.0060 (13)	0.0040 (12)	0.0039 (11)
C11	0.0149 (15)	0.0103 (14)	0.0140 (14)	0.0064 (12)	0.0050 (12)	0.0049 (11)
C12	0.0174 (16)	0.0134 (15)	0.0158 (15)	0.0080 (13)	0.0058 (13)	0.0069 (12)
C13	0.0217 (18)	0.0159 (16)	0.0185 (16)	0.0102 (14)	0.0024 (13)	0.0058 (13)
C14	0.0163 (16)	0.0148 (15)	0.0169 (15)	0.0080 (13)	0.0062 (13)	0.0062 (12)
C15	0.0136 (16)	0.0128 (15)	0.0178 (15)	0.0067 (13)	0.0024 (12)	0.0043 (12)
C16	0.0249 (18)	0.0193 (16)	0.0207 (16)	0.0112 (15)	0.0091 (14)	0.0087 (13)
C17	0.032 (2)	0.0167 (17)	0.0256 (18)	0.0128 (16)	0.0097 (16)	0.0005 (14)
C18	0.0226 (18)	0.0115 (15)	0.0364 (19)	0.0099 (14)	0.0093 (15)	0.0071 (14)
C19	0.0177 (17)	0.0171 (16)	0.0283 (18)	0.0079 (14)	0.0058 (14)	0.0122 (14)
C20	0.0185 (17)	0.0122 (15)	0.0181 (15)	0.0066 (13)	0.0042 (13)	0.0033 (12)
C21	0.0153 (16)	0.0082 (13)	0.0170 (15)	0.0063 (12)	0.0046 (12)	0.0052 (11)
C22	0.0164 (16)	0.0122 (14)	0.0193 (15)	0.0083 (13)	0.0058 (13)	0.0078 (12)
C23	0.0286 (19)	0.0157 (15)	0.0176 (15)	0.0152 (15)	0.0060 (14)	0.0062 (13)
C24	0.0246 (18)	0.0120 (15)	0.0174 (16)	0.0102 (14)	−0.0027 (14)	−0.0007 (12)
C25	0.0171 (17)	0.0145 (15)	0.0285 (18)	0.0072 (14)	0.0033 (14)	0.0060 (13)
C26	0.0208 (17)	0.0144 (15)	0.0227 (16)	0.0099 (14)	0.0095 (14)	0.0089 (13)

### Geometric parameters (Å, º)

**Table:**

Br1—C1	1.906 (3)	C12—C13	1.531 (4)
O1—N1	1.421 (3)	C12—H12	1.0000
O1—C7	1.428 (3)	C13—H13A	0.9800
N1—C8	1.281 (4)	C13—H13B	0.9800
N2—C14	1.471 (4)	C13—H13C	0.9800
N2—C10	1.477 (4)	C14—H14A	0.9800
N2—C11	1.487 (3)	C14—H14B	0.9800
C1—C2	1.386 (4)	C14—H14C	0.9800
C1—C6	1.398 (4)	C15—C16	1.385 (4)
C2—C3	1.385 (4)	C15—C20	1.394 (4)
C2—H2	0.9500	C16—C17	1.392 (4)
C3—C4	1.386 (4)	C16—H16	0.9500
C3—H3	0.9500	C17—C18	1.382 (5)
C4—C5	1.378 (4)	C17—H17	0.9500
C4—H4	0.9500	C18—C19	1.383 (4)
C5—C6	1.392 (4)	C18—H18	0.9500
C5—H5	0.9500	C19—C20	1.389 (4)
C6—C7	1.501 (4)	C19—H19	0.9500
C7—H7A	0.9900	C20—H20	0.9500
C7—H7B	0.9900	C21—C22	1.392 (4)
C8—C9	1.494 (4)	C21—C26	1.394 (4)
C8—C12	1.500 (4)	C22—C23	1.392 (4)
C9—C10	1.532 (4)	C22—H22	0.9500
C9—H9A	0.9900	C23—C24	1.384 (5)
C9—H9B	0.9900	C23—H23	0.9500
C10—C15	1.516 (4)	C24—C25	1.389 (5)
C10—H10	1.0000	C24—H24	0.9500
C11—C21	1.521 (4)	C25—C26	1.391 (4)
C11—C12	1.547 (4)	C25—H25	0.9500
C11—H11	1.0000	C26—H26	0.9500
			
N1—O1—C7	106.7 (2)	C13—C12—C11	111.2 (2)
C8—N1—O1	111.9 (2)	C8—C12—H12	107.7
C14—N2—C10	108.7 (2)	C13—C12—H12	107.7
C14—N2—C11	108.3 (2)	C11—C12—H12	107.7
C10—N2—C11	111.8 (2)	C12—C13—H13A	109.5
C2—C1—C6	122.0 (3)	C12—C13—H13B	109.5
C2—C1—Br1	118.1 (2)	H13A—C13—H13B	109.5
C6—C1—Br1	119.8 (2)	C12—C13—H13C	109.5
C3—C2—C1	119.2 (3)	H13A—C13—H13C	109.5
C3—C2—H2	120.4	H13B—C13—H13C	109.5
C1—C2—H2	120.4	N2—C14—H14A	109.5
C2—C3—C4	119.9 (3)	N2—C14—H14B	109.5
C2—C3—H3	120.1	H14A—C14—H14B	109.5
C4—C3—H3	120.1	N2—C14—H14C	109.5
C5—C4—C3	120.2 (3)	H14A—C14—H14C	109.5
C5—C4—H4	119.9	H14B—C14—H14C	109.5
C3—C4—H4	119.9	C16—C15—C20	118.6 (3)
C4—C5—C6	121.5 (3)	C16—C15—C10	120.6 (3)
C4—C5—H5	119.3	C20—C15—C10	120.8 (3)
C6—C5—H5	119.3	C15—C16—C17	120.7 (3)
C5—C6—C1	117.2 (3)	C15—C16—H16	119.7
C5—C6—C7	122.7 (3)	C17—C16—H16	119.7
C1—C6—C7	120.1 (3)	C18—C17—C16	120.3 (3)
O1—C7—C6	108.7 (2)	C18—C17—H17	119.9
O1—C7—H7A	110.0	C16—C17—H17	119.9
C6—C7—H7A	110.0	C17—C18—C19	119.6 (3)
O1—C7—H7B	110.0	C17—C18—H18	120.2
C6—C7—H7B	110.0	C19—C18—H18	120.2
H7A—C7—H7B	108.3	C18—C19—C20	120.1 (3)
N1—C8—C9	127.9 (3)	C18—C19—H19	119.9
N1—C8—C12	117.7 (3)	C20—C19—H19	119.9
C9—C8—C12	114.4 (2)	C19—C20—C15	120.7 (3)
C8—C9—C10	109.9 (2)	C19—C20—H20	119.6
C8—C9—H9A	109.7	C15—C20—H20	119.6
C10—C9—H9A	109.7	C22—C21—C26	118.0 (3)
C8—C9—H9B	109.7	C22—C21—C11	120.8 (3)
C10—C9—H9B	109.7	C26—C21—C11	121.2 (3)
H9A—C9—H9B	108.2	C21—C22—C23	121.1 (3)
N2—C10—C15	112.0 (2)	C21—C22—H22	119.5
N2—C10—C9	111.4 (2)	C23—C22—H22	119.5
C15—C10—C9	109.2 (2)	C24—C23—C22	120.2 (3)
N2—C10—H10	108.1	C24—C23—H23	119.9
C15—C10—H10	108.1	C22—C23—H23	119.9
C9—C10—H10	108.1	C23—C24—C25	119.5 (3)
N2—C11—C21	110.4 (2)	C23—C24—H24	120.3
N2—C11—C12	111.9 (2)	C25—C24—H24	120.3
C21—C11—C12	110.9 (2)	C24—C25—C26	120.0 (3)
N2—C11—H11	107.8	C24—C25—H25	120.0
C21—C11—H11	107.8	C26—C25—H25	120.0
C12—C11—H11	107.8	C25—C26—C21	121.2 (3)
C8—C12—C13	113.6 (2)	C25—C26—H26	119.4
C8—C12—C11	108.7 (2)	C21—C26—H26	119.4
			
C7—O1—N1—C8	−175.3 (2)	N1—C8—C12—C11	−123.9 (3)
C6—C1—C2—C3	−1.4 (5)	C9—C8—C12—C11	54.0 (3)
Br1—C1—C2—C3	178.8 (2)	N2—C11—C12—C8	−53.8 (3)
C1—C2—C3—C4	0.5 (5)	C21—C11—C12—C8	−177.5 (2)
C2—C3—C4—C5	0.9 (5)	N2—C11—C12—C13	−179.6 (2)
C3—C4—C5—C6	−1.5 (5)	C21—C11—C12—C13	56.7 (3)
C4—C5—C6—C1	0.6 (4)	N2—C10—C15—C16	−138.9 (3)
C4—C5—C6—C7	179.6 (3)	C9—C10—C15—C16	97.3 (3)
C2—C1—C6—C5	0.9 (4)	N2—C10—C15—C20	44.5 (4)
Br1—C1—C6—C5	−179.3 (2)	C9—C10—C15—C20	−79.3 (3)
C2—C1—C6—C7	−178.2 (3)	C20—C15—C16—C17	−0.6 (5)
Br1—C1—C6—C7	1.7 (4)	C10—C15—C16—C17	−177.2 (3)
N1—O1—C7—C6	−171.7 (2)	C15—C16—C17—C18	−0.5 (5)
C5—C6—C7—O1	−5.5 (4)	C16—C17—C18—C19	0.8 (5)
C1—C6—C7—O1	173.5 (2)	C17—C18—C19—C20	0.0 (5)
O1—N1—C8—C9	2.3 (4)	C18—C19—C20—C15	−1.2 (5)
O1—N1—C8—C12	179.8 (2)	C16—C15—C20—C19	1.4 (5)
N1—C8—C9—C10	122.8 (3)	C10—C15—C20—C19	178.0 (3)
C12—C8—C9—C10	−54.8 (3)	N2—C11—C21—C22	−72.3 (3)
C14—N2—C10—C15	61.1 (3)	C12—C11—C21—C22	52.3 (3)
C11—N2—C10—C15	−179.4 (2)	N2—C11—C21—C26	107.5 (3)
C14—N2—C10—C9	−176.3 (2)	C12—C11—C21—C26	−127.9 (3)
C11—N2—C10—C9	−56.8 (3)	C26—C21—C22—C23	−0.2 (4)
C8—C9—C10—N2	54.5 (3)	C11—C21—C22—C23	179.6 (3)
C8—C9—C10—C15	178.7 (2)	C21—C22—C23—C24	−0.7 (4)
C14—N2—C11—C21	−59.2 (3)	C22—C23—C24—C25	1.0 (4)
C10—N2—C11—C21	−179.0 (2)	C23—C24—C25—C26	−0.4 (4)
C14—N2—C11—C12	176.7 (2)	C24—C25—C26—C21	−0.5 (4)
C10—N2—C11—C12	56.9 (3)	C22—C21—C26—C25	0.8 (4)
N1—C8—C12—C13	0.5 (4)	C11—C21—C26—C25	−179.0 (3)
C9—C8—C12—C13	178.3 (3)		

### Hydrogen-bond geometry (Å, º)

Cg1 is the centroid of the C21–C26 benzene ring.

**Table:**

*D*—H···*A*	*D*—H	H···*A*	*D*···*A*	*D*—H···*A*
C4—H4···*Cg*1^i^	0.95	2.77	3.626 (4)	150

Symmetry code: (i) *x*−1, *y*−1, *z*−1.
